# Supplementary material for: Healthcare students' knowledge, attitude and perception of pharmacovigilance: A systematic review
Source: PLoS One. 2020 May 20;15(5):e0233393. doi: 10.1371/journal.pone.0233393 (PMC7239457; doi:10.1371/journal.pone.0233393)
Supplement: S1 Appendix — (DOCX) [file pone.0233393.s001.docx]

**Appendix 1. Search strategy used in MEDLINE**

| **Sr.No.** | **Search Strings** | **# of hits** |
| --- | --- | --- |
| #1 | MeSH term ‘Pharmacovigilance, Major'/exp | 1,710 |
| #2 | MeSH terms ‘Drug-Related Side Effects and Adverse Reactions' OR 'Adverse Drug Reaction Reporting Systems', major’/exp | 115,379 |
| #3 | pharmacovigilance or adverse drug reaction reporting | 15,280 |
| #4 | (pharmacovigilance or adverse drug reaction reporting' OR 'pharmacovigilance or adverse drug reaction reporting' ) (title/abstract) | 5,827 |
| #5 | #1 OR #2 OR #3 OR #4 | 121,516 |
| #6 | MeSH term "Students, Medical" OR "Students, Pharmacy" OR "Students, Nursing" OR "Students, Dental"/exp | 62,113 |
| #7 | ( medical students or medicine students or students in medicine ) OR pharmacy students OR ( nursing students or student nurses or undergraduate student nurses ) OR dental students | 194,469 |
| #8 | " medical students or medicine students or students in medicine ) OR pharmacy students OR ( nursing students or student nurses or undergraduate student nurses ) OR (dental students) "(title/abstract) | 103,258 |
| #9 | #5 OR #6 OR #7 | 194,469 |
| #10 | knowledge or attitudes or perceptions or beliefs or views or feelings or experience | 2,274,325 |
| #11 | MeSH term "Knowledge" OR "Health Knowledge, Attitudes, Practice'/exp | 113,813 |
| #12 | MeSH term "Perception'/exp | 412,407 |
| #13 | MeSH term"Attitude+" OR "Attitude to Health+" | 552,883 |
| #14 | #10 Or # 11 OR # 12 Or #13 | 2,599,368 |
| #15 | #5 and #9 and #14 | 131 |
| #16 | #15 AND English | 124 |
