# Supplementary material for: Healthcare students' knowledge, attitude and perception of pharmacovigilance: A systematic review
Source: PLoS One. 2020 May 20;15(5):e0233393. doi: 10.1371/journal.pone.0233393 (PMC7239457; doi:10.1371/journal.pone.0233393)
Supplement: S3 Appendix — (DOCX) [file pone.0233393.s003.docx]

**Appendix 3. Questions Used to Measures Knowledge, Attitude, and Perceptions of Medical Students in the Included Studies**

| **Author (year)** | **Knowledge, % Yes** | | | | | | | | | | | | | | | **Attitude, % Yes** | | | **Perceptions, % Yes** | | | | | | | | | |
| --- | --- | --- | --- | --- | --- | --- | --- | --- | --- | --- | --- | --- | --- | --- | --- | --- | --- | --- | --- | --- | --- | --- | --- | --- | --- | --- | --- | --- |
|  | PV definition | ADR definition | The important purpose of PV | Minimum information required for the ADR report submission | Local regulatory body of ADR reporting | Local reporting system of ADR reporting | The WHO online database for reporting ADR ? | It is necessary to confirm the causal relationship between the drug and the ADR | The most commonly used scales to establish the causality of an ADR | Consequence of serious ADR | ADR related to herbal products should be reported | ADR related to cosmetics and special nutritional products may be reported | Type A ADR definition | Type B ADR definition | I plan to report ADRs that I will encounter | | I'm willing to report any ADR in my future practice | Reporting of ADRs is necessary | | PV should be included in the curriculum | The topic on PV is well covered in my curriculum. | I can perform ADR reporting during the clerkship | ADR reporting should be made compulsory | ADR reporting is a professional obligation? | Information on how to report ADRs should be taught to senior students | Reporting of known ADRs makes no contribution to the reporting system | I believe that non-fatal or life-threatening ADR should not be reported. | With my present knowledge, I am well prepared to report any ADRs notice in my future practice. |
|  |  |  |  |  |  |  |  |  |  |  |  |  |  |  |  |  |  |  |  |  |  |  |  |  |  |  |  |  |
|  |  |  |  |  |  |  |  |  |  |  |  |  |  |  |  |  |  |  |  |  |  |  |  |  |  |  |  |  |
|  |  |  |  |  |  |  |  |  |  |  |  |  |  |  |  |  |  |  |  |  |  |  |  |  |  |  |  |  |
|  |  |  |  |  |  |  |  |  |  |  |  |  |  |  |  |  |  |  |  |  |  |  |  |  |  |  |  |  |
| Katyal et al (2019)[[33](#_ENREF_33)] | 60 |  |  |  |  |  |  |  |  |  |  |  |  |  |  | |  |  | |  |  |  |  |  |  |  |  |  |
| Marko (2019)[[29](#_ENREF_29)] | 54 | 60 | 60 |  | 50 | 55 | 48 |  |  |  |  |  |  |  |  | |  |  | |  |  |  |  | 70 |  |  |  |  |
| [Yu](https://onlinelibrary-wiley-com.sdl.idm.oclc.org/action/doSearch?ContribAuthorStored=Yu%2C+Yun+Mi) et al (2018)[[32](#_ENREF_32)] |  |  |  |  |  |  |  |  |  |  |  |  |  |  |  | |  |  | |  |  |  |  | 56 |  |  |  |  |
| Khan et al (2018)[[34](#_ENREF_34)] | 64 |  |  |  | 54 |  | 23 |  |  |  |  |  |  |  |  | |  |  | |  |  |  |  | 71 |  |  |  |  |
| Aamir et al (2018)[[35](#_ENREF_35)] |  |  |  |  |  |  |  |  |  |  |  |  |  |  |  | |  |  | |  |  |  |  |  |  |  |  |  |
| Gaude et al (2018)[[30](#_ENREF_30)] | 73 | 86 | 73 |  | 66 | 46 |  |  |  |  |  |  |  |  |  | |  | 97 | |  |  |  |  |  |  |  |  |  |
| Ajantha et al (2018)[[31](#_ENREF_31)] | 24 |  | 31 |  | 43 |  | 26 |  |  |  |  |  |  |  |  | |  |  | |  |  |  | 42 | 31 |  |  |  |  |
| Tadvi et al (2018)[[20](#_ENREF_20)] | 32 | 57 |  |  | 65 |  | 27 |  |  |  |  |  |  |  |  | |  | 78 | |  |  |  |  | 56 |  |  |  |  |
| Limaye et al (2018)[[38](#_ENREF_38)] |  |  |  |  |  |  |  |  |  |  |  |  |  |  |  | |  |  | | 68 | 21 |  | 74 |  |  |  | 77 |  |
| Chhabra et al (2017)[[24](#_ENREF_24)] |  |  |  |  |  |  |  |  |  |  |  |  |  |  |  | |  |  | |  |  |  |  |  |  |  |  |  |
| Alkayyal et al (2017)[[16](#_ENREF_16)] | 16 | 69 |  |  | 60 |  |  |  |  |  | 14 |  |  |  |  | | 80 |  | |  | 79% |  | 69 |  | 82 |  | 27 | 44 |
| Othman et al (2017)[[39](#_ENREF_39)] |  |  |  |  |  |  |  |  |  |  |  |  |  |  |  | |  |  | | 9 |  |  | 96 |  |  |  | 61 | 84 |
| Al-Shekaili et al (2017)[[17](#_ENREF_17)] | 61 | 56 |  |  | 78 | 72 | 17 |  |  |  |  |  | 63 | 51 |  | |  |  | | 81 | 71 | 65 | 61 |  |  | 20 |  | 48 |
| Osemene et al (2017)[[15](#_ENREF_15)] | 51 | 49 |  |  | 48 | 42 |  |  |  |  |  |  |  |  |  | |  |  | |  |  |  |  |  |  |  |  |  |
| Schutte et al (2017)[[21](#_ENREF_21)] |  |  |  |  |  |  |  |  |  |  | 53 | 34 |  |  |  | |  |  | | 79 | 26 | 38 |  |  |  | 14 | 1 | 30 |
| Rajiah et al (2016)[[18](#_ENREF_18)] |  |  |  |  |  |  |  |  |  |  |  |  |  |  |  | |  |  | |  |  |  | 90 |  | 96 |  | 47 |  |
| Abubakar et al (2015)[[22](#_ENREF_22)] | 66 | 80 |  |  |  |  |  |  |  |  |  |  |  |  |  | |  |  | |  |  |  |  | 82 | 82 |  |  |  |
| Farha et al (2015)[[27](#_ENREF_27)] | 24 | 62 |  |  | 29 | 24 |  | 79 |  |  |  |  |  |  |  | |  |  | |  | 85 | 65 | 73 |  | 84 | 36 | 22 |  |
| Kothari et al (2015)[[28](#_ENREF_28)] | 45 |  |  |  | 19 |  |  |  |  |  |  |  |  |  |  | |  |  | |  |  |  | 68 |  |  |  |  |  |
| Khan et al (2015)[[26](#_ENREF_26)] | 31 | 84 |  | 42 | 37 |  |  |  |  |  |  |  | 40 | 36 |  | |  |  | | 88 |  |  | 97 |  |  |  |  |  |
| Shalini et al (2015)[[25](#_ENREF_25)] | 25 | 57 |  |  | 0 |  |  |  |  |  |  |  |  |  |  | |  |  | |  |  |  |  |  |  |  |  |  |
| Jha et al (2014)[[40](#_ENREF_40)] |  |  |  |  |  |  |  |  |  |  |  |  |  |  |  | |  |  | |  |  |  |  |  |  |  |  |  |
| Sivadasan et al (2014)[[12](#_ENREF_12)] | 47 | 72 | 57 |  | 13 | 6 | 8 |  | 5 |  |  |  |  |  |  | |  |  | | 79 | 24 |  | 79 | 84 | 88 |  |  | 40 |
| Reddy et al (2014)[[14](#_ENREF_14)] | 77 |  | 62 |  | 62 |  | 25 |  |  |  |  |  |  |  |  | |  |  | |  |  |  |  | 40 |  |  |  |  |
| Sivadasan et al (2014)[[23](#_ENREF_23)] | 50 | 38 | 19 |  | 9 | 6 | 53 |  | 31% |  |  |  |  |  |  | |  |  | | 67 | 82 |  | 56 | 72 |  | 64 |  | 82 |
| Sharma et al (2012)[[13](#_ENREF_13)] |  |  |  |  |  |  |  |  |  |  |  |  |  |  |  | |  |  | | 73 | 17 |  | 80 |  | 70 | 17 | 19 | 17 |
| Gavaza et al. (2012)[[19](#_ENREF_19)] |  |  |  |  |  |  |  |  |  |  |  | 78 |  |  |  | |  |  | |  |  |  |  |  |  |  |  |  |
| Elkalmi et al (2011)[[11](#_ENREF_11)] | 61 | 76 |  | 40 | 94 |  |  | 18 |  | 87 | 31 |  | 77 | 60 |  | |  |  | | 58 |  |  |  |  |  |  |  |  |
| Kalari et al. (2011)[[36](#_ENREF_36)] | 32 | 57 |  |  | 65 |  | 27 |  |  |  |  |  |  |  |  | |  | 78 | |  |  |  |  | 56 |  |  |  |  |
