# Supplementary material for: Healthcare students' knowledge, attitude and perception of pharmacovigilance: A systematic review
Source: PLoS One. 2020 May 20;15(5):e0233393. doi: 10.1371/journal.pone.0233393 (PMC7239457; doi:10.1371/journal.pone.0233393)
Supplement: Attachment — Submitted filename: Response_reviewer_R2.docx [file pone.0233393.s005.docx]

We would like to thank the editor and the reviewers for the time and effort they spent on reviewing our revised manuscript entitled **“Healthcare Students' Knowledge, Attitude and Perception of Pharmacovigilance: A Systematic Review"**

All appropriate changes/amendments have been made correspondingly to the paper (Highlighted) are summarized in the following:

**Reviewer Comments to Authors**

**Comment #** 1:   *The search strategy is improved. However, if you did not have the searches developed and carried out in collaboration with a trained information specialist please add this to the limitations of the study, as you cannot be certain that there were not additional studies that you did not retrieve.*

**Response:** Thank you for the suggestion; we have added this limitation to page 12, lines 1-2

*Also, did you search the Cochrane Database of Systematic Reviews or was it the Cochrane Library? If you were searching for systematic reviews it was probably the Cochrane Database of Systematic Reviews but if you were searching for individual controlled trials it was probably the Cochrane Central Register of Controlled Trials. Both of these databases are in the Cochrane Library.*

**Response:** We have searched the *Cochrane Database of Systematic Review (Page 6, line 3).*

**Comment #** 2:   *The rationale for including a ‘pilot study’ in which pre and post tests were administered and excluding studies reporting KAP measures pre- and post- some intervention is unclear. What is the difference between assessing the results of an intervention and testing the effects of usual education? This needs to be clarified.*

**Response:** The reason for excluding interventional studies was because we are not evaluating the impact of educational material on knowledge, attitude, and perceptions. The reason for including pilot studies, because these studies are usually conducted to evaluate the validity, readability, and reliability of the instruments used in the survey usually among 20-30 participants (Results from the pilot subjects are not included in the study). For example, Limaye et al. study was pilot-tested to a sample of 30 pharmacy students (Pilot subjects (i.e., 30) were not part of the final study that was conducted among 352 students).

*The inclusion of cross-sectional studies is clear, but the cohort studies could mean anything from a single group tested at multiple time points to a randomized controlled trial.*

**Response:** We totally agree with the reviewer, therefore we have removed cohort study from the inclusion criteria.

**Comment #** 3:  *The reviewer is correct that STROBE is a reporting guideline. The authors of the article you cite say that STROBE was referred to in many of the assessment instruments because many STROBE items may have been selected because of presumed association with risk of bias. However the authors do not suggest that STROBE should be used as a risk of bias assessment tool. The authors state that ‘Just under three-quarters of all tools were proposed as being suitable for future use, including all of the critical appraisal tools and generic systematic review tools and six of the tools originally designed for use in a specific systematic review.’ Look in Tables 5-7 for the tools that meet the important criteria identified by the authors (selection of participants, measurement of variables, and control of confounding). These are the tools that the authors judge as appropriate.
A more recent and more targeted approach to risk of bias for surveys may be found in* [*https://www.evidencepartners.com/wp-content/uploads/2017/09/Risk-of-Bias-Instrument-for-Cross-Sectional-Surveys-of-Attitudes-and-Practices.pdf*](https://www.evidencepartners.com/wp-content/uploads/2017/09/Risk-of-Bias-Instrument-for-Cross-Sectional-Surveys-of-Attitudes-and-Practices.pdf)*.*

*See* [*https://www.evidencepartners.com/wp-content/uploads/2017/04/Methods-Commentary-Risk-of-Bias-in-cross-sectional-surveys-of-attitude....pdf*](https://www.evidencepartners.com/wp-content/uploads/2017/04/Methods-Commentary-Risk-of-Bias-in-cross-sectional-surveys-of-attitude....pdf) *for comments and further guidance on this tool.*

**Response:** We thank the reviewer for the detailed explanation about this important point and for providing the reference. Now, we have incorporated under the quality assessment the five items risk of bias in cross-sectional surveys of attitudes and practice (Page 7, lines 7-8) (Page 8, lines 15-16).

**Reference:** Agarwald, A., G. Guyatt, and J. Busse. "Methods commentary: Risk of bias in cross-sectional surveys of attitudes and practices." (2019).

*For the pre-post surveys, you might have to look further for a suitable tool, or you could decide to assess the risk of bias based on either the pretest or the posttest survey if you are not interested in the effects of the education intervention. However, as I mentioned above, the inclusion of pretest/posttest pilot studies but the exclusion of other interventional studies lacks a rationale and this needs to be addressed.*

**Response:** Response to this comment was hopefully explained in response to the second comment.
